# Short- and Long-Term Dynamics of Endothelial Cell Density and Vault Changes After Implantable Collamer Lens Implantation

**DOI:** 10.3390/medsci14060656

**Published:** 2026-10-09

**Authors:** Maria Muzyka-Woźniak, Adam Oleszko, Malwina Terlecka

**Affiliations:** 1 Research and Development Center CREO, Clinical Ophthalmology Center SPEKTRUM, Zaolzianska 4, 53-334 Wroclaw, Poland; 2 Department of Fundamental Problems of Technology, Wroclaw University of Science and Technology, Wybrzeze Wyspianskiego 27, 50-370 Wroclaw, Poland; adam.oleszko@pwr.edu.pl (A.O.); malwina.terlecka@pwr.edu.pl (M.T.)

**Keywords:** implantable collamer lens, postoperative vault, endothelial cell loss, phakic intraocular lens implantation

## Abstract

**Background/Objectives**: Endothelial cell density (ECD) and vault height are essential safety metrics in implantable collamer lens (ICL) surgery, yet their reported decay rates vary. This study assessed short- and long-term changes in ECD and vault height. **Methods**: Medical records of patients undergoing V4c ICL implantation between 2014 and 2024 were reviewed. ECD loss was evaluated across three periods: early (baseline to 6 months), late (6 months to last visit), and total (baseline to last visit). Vault was measured at day 1 and months 3, 6, 12, and the last visit and decay rate was calculated for the entire follow-up period. **Results**: A total of 631 eyes of 323 patients were analyzed. The early, late, and total ECD losses per year were 1.81 ± 8.56% (95% CI 0.43 to 3.19), 0.68 ± 2.11% (95% CI 0.34 to 1.03), and 1.21 ± 2.58% (95% CI 0.83 to 1.59), respectively. The ECD loss rate was not correlated with vault. Vault decreased rapidly during the first 3 postoperative months (−178.0 µm/year; 95% CI, −211.4 to −144.6; *p* < 0.001), followed by a substantially slower but still significant decline (−19.1 µm/year; 95% CI, −23.9 to −14.2; *p* < 0.001). The difference between the early and late slopes was significant (*p* < 0.001). The vault decay rate was 6.80 ± 10.41% (95% CI, 5.48 to 8.13%) per year and was not associated with ICL size or anterior chamber depth. **Conclusions**: ECD loss was greatest and most variable during the first 6 months postoperatively. Thereafter, a slower sustained decline was observed. Vault decay showed a biphasic pattern, with a relatively rapid decrease during the first 3 postoperative months and a significantly slower long-term decline.

## 1. Introduction

Posterior chamber phakic implantable collamer lenses (ICL; STAAR Surgical) are increasingly used for the surgical correction of refractive errors. Long-term safety is assessed mainly in relation to adequate ICL vaulting and the impact of ICL presence in the eye on the corneal endothelium. Published long-term data on changes in corneal endothelial cell density (ECD) after ICL implantation remain inconsistent: some studies report no significant ECD change [1,2], whereas others describe significant loss, with mean annual rates ranging from 0.98% to 3.6% [3,4,5,6,7,8]. It also remains unclear whether the observed decrease in ECD should be attributed primarily to the surgical procedure or to other chronic factors [7,9,10,11]. The surgical intervention itself is estimated to induce a mean ECD reduction of 1.32% ± 1.28% relative to the preoperative baseline [11]. However, published studies do not consistently distinguish the early postoperative decrease in ECD attributable to surgical trauma from the subsequent long-term decline, and frequently report cumulative endothelial cell loss. At 12 months postoperatively, the reported cumulative reduction in ECD was 1.75% ± 2.17%, exceeding the expected physiological rate of age-related corneal endothelial cell attrition [11]. In clinical practice, understanding the dynamics of ECD change might help optimize follow-up schedules and define age- and life expectancy-adjusted ECD cut-off values for exclusion criteria.

Similar uncertainty applies to postoperative vault reduction. It is generally accepted that the optimal vault ranges between 250 and 750 microns (μm). The excessively high vault (over 1000 μm) may cause angle crowding and interfere with the pupil dynamics, while the low vault (less than 100 μm) increases the risk of crystalline lens contact and cataract formation [8,12,13]. Although all 10-year follow-up studies have reported a decrease in vault, the pattern of early and later postoperative vault changes remains insufficiently characterized [1,2,4,6,7]. A detailed analysis of these changes is required to guide timely management of both low and high vault.

This study aimed to evaluate the dynamics of ECD decline and vault change over a 10-year follow-up period after ICL V4c implantation and to estimate annual decay rates.

## 2. Materials and Methods

### 2.1. Study Design and Data Collection

Medical records of patients who underwent V4c ICL implantation at a single ophthalmology centre between December 2014 and September 2024 were reviewed. The analysis included ICL models VICM0, VICTM0, VICM5, and VICTM5; eyes implanted with hyperopic ICLs were excluded. Inclusion criteria were age of at least 21 years and preoperative ECD of at least 2000 cells/mm^2^. The exclusion criteria included any previous ocular surgery, history of inflammation or trauma and insufficient follow-up duration. The preoperative and postoperative data extending up to 10 years were collected and changes over the available observation time were analyzed. The minimal follow-up period required was 12 months. 

The preoperative biometric parameters were recorded: axial length (AL), pupil diameter (PD) and keratometry obtained with IOLMaster 700 or IOLMaster 500 (Carl Zeiss Meditec, Jena, Germany); anterior chamber depth (ACD) measured from the corneal endothelium to the anterior lens with anterior segment optical coherence tomography (AS-OCT) Visante (Carl Zeiss Meditec, Jena, Germany) or Casia 2 (Tomey Corporation, Nagoya, Japan); and horizontal white-to-white distance (WTW) and central corneal thickness (CCT) measured with Pentacam AXL (Oculus Optikgeräte GmbH, Wetzlar, Germany). Postoperative assessments of central ECD and vault were recorded on day 1; at months 1, 3, 6, and 12; and annually thereafter. Dilated fundus examination and slit-lamp assessment for lens opacity were also performed. The incidence of adverse events such as cataract formation, glaucoma, retinal detachment, and severe endothelial loss was evaluated. 

The study was conducted in accordance with the Declaration of Helsinki and the Declaration of Taipei on Ethical Considerations regarding Health Databases and Biobanks and approved by the Ethics Committee of Lower Silesia Medical Council (04/BNR/2026, 8 July 2026). The analysis was based exclusively on previously collected, fully anonymized records and did not entail patient contact or new data collection.

### 2.2. ICL Selection and Surgical Procedure

The ICL power and size calculation was performed using the OCOS algorithm provided by the manufacturer (Staar Surgical, Nidau, Switzerland). Only eyes targeted for emmetropia were included in the analysis. All surgeries were performed by three experienced surgeons, with a temporal corneal incision of 3.0 mm. The anterior chamber was filled with 2% hydroxypropyl methylcellulose (AJL CELL, BAUSCH & LOMB GmbH, Berlin, Germany) and the ICL was implanted using the injector cartridge system (Staar Surgical, Nidau, Switzerland). The viscosurgical device was washed out using buffered salt solution and corneal incisions were hydrated. Myotic agents were not administered upon conclusion of the surgical procedure.

### 2.3. Corneal Endothelial Cell Density

Central corneal ECD was assessed with a non-contact specular microscope (Topcon, SP-3000P, Topcon Corporation, Tokyo, Japan). At least 100 cells were marked and manual correction of automated cell recognition was performed for each measurement. At least 2 central measurements were performed for each visit and the mean value was calculated. Only high-quality images were included. The ECD loss was analyzed across three intervals: (1) early, from preoperative baseline to 6 months; (2) late, from 6 months to the last visit; and (3) total, from preoperative to the last assessment. ECD loss was calculated with the following formula, adopted from Packer et al. [5]:

(1) $$\text{ECD loss early}\;=\;\frac{\text{ECD preop}\;-\;\text{ECD}\;6m}{\text{ECD preop}},$$

(2) $$\text{ECD loss late}\;=\;\frac{\text{ECD}\;6m\;-\;\text{ECD last}}{\text{ECD}\;6m},$$

(3) $$\text{ECD loss total}\;=\;\frac{\text{ECD preop}\;-\;\text{ECD last}}{\text{ECD preop}},$$

Additionally, for a subset of eyes with ECD measured 1 month after surgery, the immediate postoperative loss was calculated similarly. Monthly and annual rates were calculated for each ECD loss interval. 

To ensure consistency across longitudinal comparisons and to minimize bias arising from missing observations, the analyses of early, late, and total ECD loss rates included only eyes for which complete ECD measurements were available at every prespecified follow-up time point required for the corresponding analysis. 

### 2.4. Vault

Vault height was measured along the visual axis using AS-OCT (Visante till August 2022, Casia 2 from August 2022). All examinations were performed under photopic conditions without pupil dilation, using a horizontal scan. For Visante images, measurements were obtained manually with digital callipers; for Casia 2, the posterior surface of the phakic intraocular lens (pIOL-B) and the anterior surface of the crystalline lens (Lens-F) were manually adjusted on the 2D CCT/ACD scan. For vault assessment, the following time points were set: 1 day, 3 months, 6 months, 12 months and last visit. 

A vault decay rate was calculated as

(4) $$\text{vault decay}\;=\;\frac{\text{vault day}\;1\;-\;\text{vault last}}{\text{vault day}\;1}\;÷\;\frac{n}{12},$$

where n equals total months of observation.

To ensure methodological continuity during the transition from the Visante AS-OCT to the CASIA2 AS-OCT system (August 2022), ACD and vault measurements were obtained using both devices during the crossover period. Comparative analysis revealed no statistically significant device-related differences in either ACD or vault measurements, indicating that the change in imaging platform was unlikely to have introduced a systematic measurement bias or materially affected the longitudinal comparability of these parameters.

### 2.5. Statistics

Statistical analyses were performed using SPSS Statistics 23.0 (SPSS Corp., Armonk, NY, USA), Python 3.11 with statsmodels library and StatPlus PRO v7.8.4 for Excel. Eyes that required any secondary intervention after ICL implantation were excluded from the ECD and vault analyses. Data normality was assessed with the Kolmogorov–Smirnov test. Longitudinal vault data were analyzed using linear mixed-effects models (LMM) that account for repeated measurements and inter-eye dependence, while multivariable analyses of vault decay and ECD loss were performed using generalized estimating equations (GEEs) with patient-level clustering and robust covariance estimates.

Correlation analyses were performed using rank-based correlation coefficients. Because both eyes of the same patient could be included, statistical inference was adjusted for within-patient clustering. For each pair of variables, both variables were rank-transformed and standardized. A GEE model was then fitted, with patient ID as the clustering variable, an independence working correlation structure, and robust sandwich standard errors. The resulting regression coefficient corresponded to the rank-based association, while the robust standard error was used to derive 95% confidence intervals and *p*-values accounting for inter-eye dependence. Analyses were performed using all available paired observations for each variable pair. To account for multiple testing, *p*-values were adjusted using the Holm procedure within each prespecified family of correlations. A two-sided *p* value < 0.05 after adjustment was considered statistically significant. 

## 3. Results

### 3.1. Demography

In total, 631 eyes of 323 patients (246 women) were analyzed. The mean patient age at the time of implantation was 31.7 ± 7 years (range: 21–53 years). Myopic Toric ICLs (models VITCMO/VITCM5) were implanted in 441 eyes (70%), and myopic ICLs (models VICMO/VICM5) were implanted in 190 eyes (30%). ICL sizes 12.1, 12.6. 13.2 and 13.7 were implanted in 19 (3%), 195 (31%), 379 (60%) and 38 (6%) eyes, respectively. The preoperative biometric data are presented in Table 1.

### 3.2. Endothelial Cell Density

The early, late and total ECD loss rates were calculated for the subset of 176 eyes (95 patients) with available data at all predetermined time points and a total follow-up time of 40 ± 21 months (range: 12 to 92 months) (Table 2). The preoperative biometric data for this subset did not differ systematically from the eyes with incomplete measurements (Supplementary Table S1). The ECD significantly declined from preoperative baseline to 6 months postoperative (Holm-adjusted *p* = 0.014) and to the last visit (Holm-adjusted *p* < 0.0001) based on an LMM accounting for within-patient clustering (Table 2). The ECD loss rate during the first 6 months postoperatively was approximately 3 times larger than in the following time period, but its confidence interval was also the widest, reflecting the greater variability of this parameter. In the subset of 106 eyes with 1-month data, the log-transformed LMM estimated a 0.38% ECD reduction during the first postoperative month (95% CI, −0.45% to 1.20%; *p* = 0.366). The maximal percentage ECD loss was 14.67, 18.28 and 23.45% for early, late and total follow-up periods.

After adjustment for within-patient clustering and multiple comparisons, baseline ECD showed a weak positive correlation with the early annualized ECD loss rate (ρ = 0.211; clustered 95% CI, 0.071–0.351; Holm-adjusted *p* = 0.029). This finding warrants caution because baseline ECD is incorporated into the calculation of early ECD loss. Early and late annualized ECD-loss estimates were inversely correlated (ρ = −0.359; clustered 95% CI, −0.492 to −0.226; Holm-adjusted *p* < 0.001), although this relationship may partly reflect the shared 6-month ECD measurement used to derive both estimates.

No consistent independent association was observed for age, ACD, CCT, preoperative spherical equivalent, ICL size or initial vault with early, late, or total annualized ECD loss. For late ECD loss, greater WTW was independently associated with a lower ECD loss rate (β = −2.67 percentage points/year per mm; 95% CI, −4.71 to −0.63; *p* = 0.01). 

### 3.3. Vault

**Figure 1:**
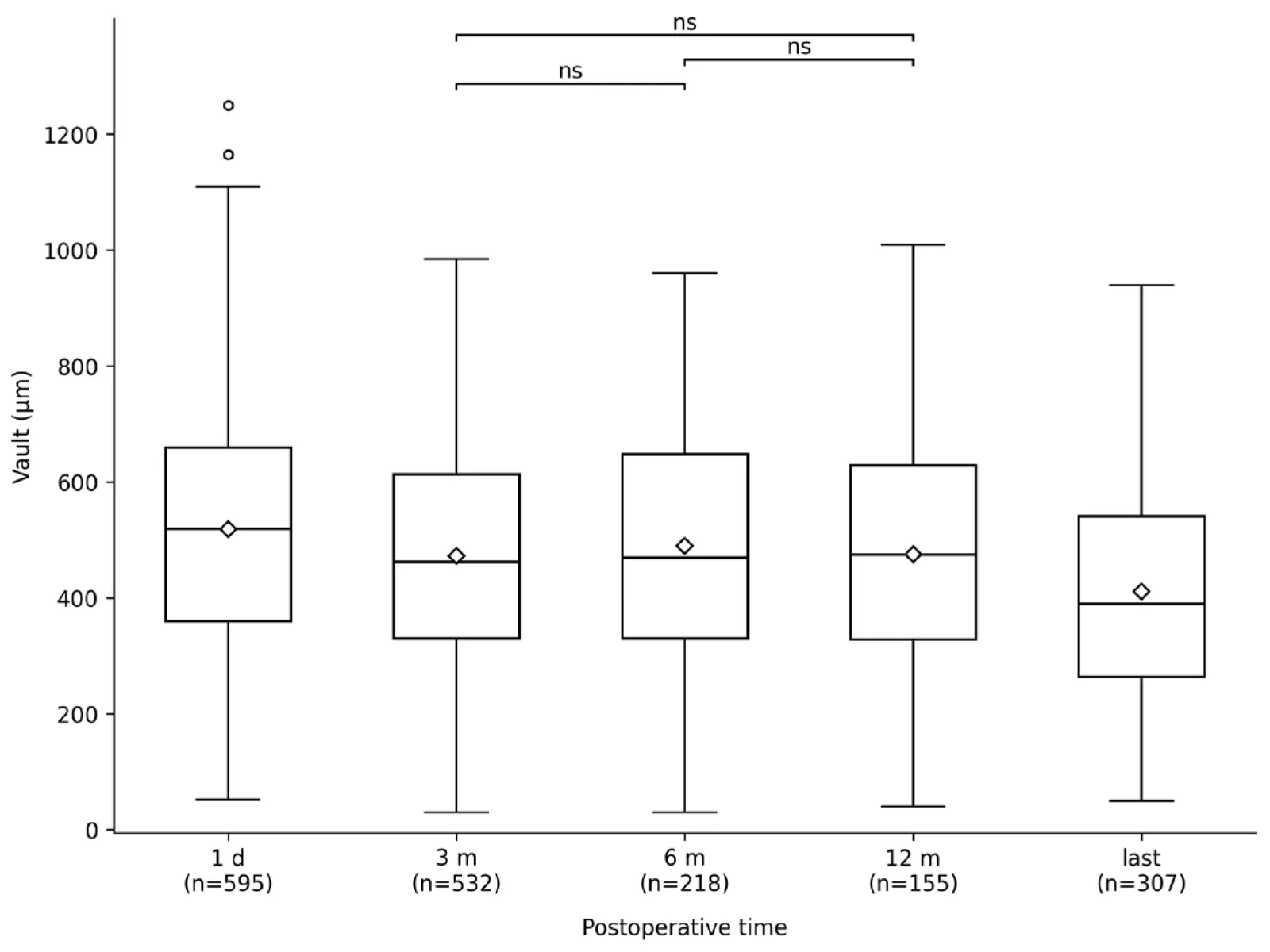
Change in mean vault height throughout the entire follow-up period. Box plot whiskers are extended to 1.5 × the interquartile range (Tukey definition). Dots represent mild outliers, and diamonds represent mean values. Except for the comparisons between 3, 6, and 12 months, all other pairwise comparisons were statistically significant (*p* < 0.0001, mixed-effects model comparisons with Holm-adjusted *p*-values). ns = non significant.

The long-term vault data were available for 307 eyes with an observation time of 39 ± 21 months (range: 14 to 108 months). The mean vault values at day 1, 3 months, 6 months, 12 months and last visit were 518 ± 218 μm, 472 ± 194 μm, 494 ± 204 μm, 478 ± 198 μm and 410 ± 183 μm, respectively (Figure 1). The vault at day 1 was significantly higher than at 3, 6, and 12 months and at the last visit (adjusted *p* < 0.0001). No statistically significant differences were observed between 3 and 6 months (adjusted *p* = 0.053), 3 and 12 months (adjusted *p* = 0.969), or 6 and 12 months (adjusted *p* = 0.182). In contrast, the last-visit vault was significantly lower than the 3-, 6-, and 12-month values. Because the postoperative vault trajectory showed different early and later dynamics, time was modelled using a piecewise linear function with a breakpoint at 3 months. Vault decreased rapidly during the first 3 postoperative months (−44.5 µm corresponding to −178.0 µm/year; 95% CI, −211.4 to −144.6; *p* < 0.001), followed by a substantially slower but still significant decline thereafter (−19.1 µm/year; 95% CI, −23.9 to −14.2; *p* < 0.001). The difference between the early and late slopes was statistically significant (*p* < 0.001). A weak positive correlation was observed between vault height at day 1 and ACD (ρ = 0.267, clustered 95% CI 0.081 to 0.454, Holm adjusted *p* = 0.015). Initial vault was not significantly associated with early, late or late annualized ECD loss after accounting for within-patient clustering and multiple comparisons (all Holm-adjusted *p* ≥ 0.589).

The vault decay rate was 6.80 ± 10.41% with a patient clustered 95% CI of 5.48 to 8.14% per year. No independent association was detected between vault decay rate and age, CCT, WTW, ACD, or preoperative baseline ECD or ICL size. In the patient clustered multivariable GEE model, preoperative absolute spherical equivalent was independently associated with annual vault decay (β = 0.58 percentage points/year per diopter; 95% CI, 0.12 to 1.04; *p* = 0.014). A weak positive correlation between the initial vault (day 1) and decay rate was observed (ρ = 0.159, clustered 95% CI 0.081 to 0.454; Holm-adjusted *p* < 0.016).

### 3.4. Adverse Events

Adverse events were evaluated in the entire cohort throughout follow-up. Cataracts occurred in two cases (0.3%): one anterior subcapsular cataract and one nuclear cataract. The localized anterior subcapsular cataract developed within the first postoperative week and was most likely related to intraoperative manipulation in one of the initial cases; it was also associated with low vault (160 μm on day 1 and 80 μm at 9 years). After 9 years of follow-up, the best corrected visual acuity was 0.1 logMAR and no surgical intervention was decided till the time of publication. The nuclear cataract developed in a 40-year-old patient with high preoperative myopia and astigmatism (−17.0 D sph/−3.0 D cyl) and normal vault (560 μm). The uneventful simultaneous ICL explantation and cataract phacoemulsification were performed 8 years after ICL implantation.

Seven secondary surgical interventions were performed (1.08%). These included two cases of ICL replacement for high vault (0.3%), two cases of ICL replacement for low vault (0.3%), two cases of surgical rotation of non-toric ICLs to an oblique position because of high vault (0.3%), and one case of ICL replacement with a larger size lens (13.7 mm) due to recurrent toric ICL autorotation despite normal vault (309 μm). 

No retinal detachment occurred in the cohort. Myopic choroidal neovascularization developed bilaterally in one patient (0.15%) and was treated with intravitreal anti-VEGF injections. In one eye, ECD decreased from 2557 cells/mm^2^ preoperatively to 1854 cells/mm^2^ at 6 months and 1564 cells/mm^2^ at 40 months, despite the absence of intraoperative complications and a normal vault. The patient is monitored every 6 months. 

## 4. Discussion

This longitudinal observational study focused on ECD and vault change dynamics after ICL implantation. The greatest ECD loss occurred during the first 6 postoperative months, followed by a slower long-term decline. The annualized ECD loss rate was also more variable during this early period than during later follow-up. Vault decreased rapidly during the first 3 months after implantation. After the ICL position had stabilized, vault decreased substantially slower but still significantly over time.

Both ECD and vault height are associated with significant measurement variability, depending on multiple factors. However, they represent important safety markers for ICL implantation. Human ECD undergoes a continuous, age-related decline beginning early in life. This physiological attrition reflects the limited proliferative capacity of corneal endothelial cells and progresses at an average rate of approximately 0.6% per year, resulting in a gradual reduction in the endothelial reserve over the lifespan [14,15]. Iris-fixated anterior chamber phakic IOLs (pIOLs) can cause significant acceleration of ECD decline, raising concerns for corneal safety [16]. Posterior chamber phakic lenses, which are positioned behind the iris and farther from the corneal endothelium, are considered a safer alternative in this regard. The Food and Drug Administration (FDA) approval for the ICL includes preoperative age-based ECD recommendations [17], although, in practice, they are not being followed by surgeons [5,10]. The German Ophthalmology Society guidelines for ICL surgery set the preoperative ECD at 2000 cells/mm^2^, irrespective of the patient’s age [18].

The published ECD loss estimates refer to different follow-up periods and are based on different methods of calculating ECD decline, using either preoperative or postoperative ECD as the baseline and reporting either cumulative loss over the entire observation period or annualized loss rates [3,4,5,6,7]. While cumulative estimates do not capture the temporal pattern of ECD decline, annualized rates allow comparisons across studies. Long-term reports indicated an average annual ECD loss of 0.98–1.2% after ICL implantation [6,7], which is consistent with the total yearly ECD loss of 1.21 ± 2.58% calculated in the present study. 

However, published data do not clearly indicate when ECD loss occurs, as assessment time points vary across studies. In the FDA ICL trial, the largest decrease was observed within the first 3 years after implantation [9]. A more recent study reported a 1.02 ± 4.32% ECD loss between preoperative and 2–3-week postoperative measurements [7], whereas another found no difference between preoperative and 3-month postoperative ECD values [10]. A recent meta-analysis of published data suggested that the ECD loss after V4C implantation is most pronounced 3 months after surgery [11]. In the present study, the greatest ECD decrease occurred during the first 6 postoperative months, exceeding the estimated physiological ECD decline by a factor of 3. Thereafter, ECD loss only slightly exceeded the expected physiological decline. This pattern may reflect compensatory remodelling of the corneal endothelium after surgical trauma. This process begins within days through cell migration and enlargement, progresses over several weeks with cytoskeletal and junctional reorganization, and reaches structural stability at approximately 3–6 months [19].

Nevertheless, the long-term variability in ECD loss should be considered, as maximum values may reach clinically relevant levels. We were unable to identify any true risk factors or predictors for larger ECD loss, which could help to predict cases requiring closer monitoring. The observed association between greater WTW and a lower annualized ECD loss rate may reflect differences in ocular anatomy and surgical geometry. A larger WTW may be associated with a larger anterior segment, potentially providing greater working space and reducing mechanical or fluid-related stress on the corneal endothelium during surgery. However, WTW may also serve as a marker of other ocular anatomical characteristics that were not fully captured in the analysis. Therefore, this association should not be interpreted as evidence of a direct protective effect of WTW on endothelial cell survival.

The weak positive correlation between early ECD loss and preoperative ECD, also reported by other researchers [7], may reflect the characteristics of counting statistics, with a greater number of cells being sampled in high-ECD corneas. Therefore, this association should not be regarded as clinically meaningful. 

The adequate vault remains the primary safety concern. Despite numerous sizing methods and the incorporation of artificial intelligence, there is still some uncertainty regarding proper ICL diameter selection. In cases presenting with postoperative vault values that are either excessively high or insufficient, determining whether and when to explant the ICL becomes a pivotal clinical decision. This consideration is further complicated by the well-documented tendency for vault height to progressively decrease over time, influenced by factors such as crystalline lens growth and postoperative anatomical remodelling [6,13]. Consequently, characterizing the temporal dynamics of vault change is essential, as it directly informs risk stratification, follow-up intervals, and the threshold at which intervention becomes advisable. 

Many studies have investigated long-term vault decay [1,2,3,4,6,20]; however, data on early vault changes remain limited. Our findings indicate that early postoperative vault is highly variable during the early postoperative period, with rapid decrease occurring during the first three months. Once the ICL stabilizes, vault declines substantially more slowly, and further significant changes become apparent only over longer follow-up intervals. This slow reduction is biologically plausible and may result from progressive crystalline lens growth or continued settling of the ICL within the ciliary sulcus. Together, these findings underscore the need to distinguish early, rapid postoperative vault decrease from the later, much slower phase of vault evolution. Clinically, this supports close monitoring of extreme vault values during the first 3 months postoperatively, when vault may still undergo substantial changes.

The long-term vault decay rate in our cohort was slightly higher than that reported in a Korean population (6.8% vs. 5.6% per year) [7]. This difference may reflect the larger anterior chamber dimensions, the correspondingly larger implanted ICL sizes, and the broader inclusion criteria in the present study, including a predominance of toric ICLs. Despite these anatomical and procedural differences, our findings are consistent with those of Chung et al., showing no significant association between vault decay rate and ICL size. However, our weak positive association between initial vault and vault decay rate differs from the weak negative correlation reported by Chung et al. [7] and supports the tendency reported by Li et al. toward greater vault reduction in eyes with higher vault [13]. These differences may be highly dependent on cohort characteristics, ICL size distribution, and ocular anatomy. The discrepancy may also be related to differences in follow-up schedules: the cited studies used the 1-month vault as a baseline with more dispersed follow-up visits, whereas our study used day-1 vault and closer observation during the first postoperative year. We believe that such a tighter monitoring scheme in the first postoperative period is more important from the point of view of clinical decisions and easier to apply in the context of patient reporting for check-ups.

Choi et al. reported an association between high vault and ECD loss rate in a bilateral paired-eye comparison; however, the main effect of vault group alone was not significant [21]. This is consistent with the absence of a significant correlation between either early or late ECD loss and vault height in our cohort.

Interestingly, we observed an association between preoperative absolute spherical equivalent and the rate of vault decay. This association may reflect larger anterior-segment dimensions in highly myopic eyes, which could provide more space for the ICL to settle. However, neither ACD nor WTW was associated with vault decay, weakening support for this proposed mechanism. Therefore, this finding should be interpreted cautiously and requires further investigation. 

The adverse event frequency in this cohort was low and comparable to other reports [4,17]. The low cataract incidence further supports the protective role of the central port against cataract formation [22]. The only case of anterior subcapsular cataract was associated with low vault and was most likely related to the early surgical learning curve. Marked ECD loss was observed in only one eye, with no identifiable associated factors. No eye showed an ECD below 1500 cells/mm^2^ during the entire follow-up period. The low number of secondary interventions supports the long-term viability of ICL for myopia and astigmatism correction. 

An important strength of our study is that both vault and ECD measurements were performed during the early postoperative period, whereas assessments in previous studies were less frequent or began later [2,4,7,13]. This provides valuable insight into the early dynamics of ICL safety parameters and may inform clinical decision-making and follow-up scheduling.

The main limitation of this study is its retrospective design, which prevented complete data collection for all patients throughout follow-up. Second, vault was measured using the Visante system until 2022 and the CASIA2 system thereafter; however, both AS-OCT devices use similar technology, and no significant differences in anterior chamber measurements have been reported [23]. Although all vault measurements were performed under standardized conditions by two experienced examiners, intra- and interobserver reproducibility was not formally assessed. Therefore, measurement variability cannot be fully excluded and should be considered when interpreting the longitudinal changes in vault.

## 5. Conclusions

ECD loss was highest during the first 6 months postoperatively. The mean annual ECD loss rate showed greater variability during the first 6 months than in the later postoperative period. Vault changes also demonstrated a biphasic pattern: a relatively rapid decrease during the first 3 months after implantation, followed by a substantially slower long-term decline.

## Figures and Tables

**Table 1:** Preoperative biometric data.

Preoperative Biometric Data				
Parameter ^1^	Mean	SD	Min	Max
AL (mm)	26.55	1.37	23.28	32.73
ACD (mm)	3.29	0.24	2.77	4.06
CCT (μm)	535	37	425	653
PD (mm)	4.9	1.1	2.7	8.4
WTW (mm)	11.8	0.4	10.9	12.8
Keratometric astigmatism (D)	1.35	0.85	0.17	4.30
ECD (cells/mm^2^)	2796	295	2015	3621

^1^ AL = axial length; ACD = anterior chamber depth; CCT = central corneal thickness; PD = pupil diameter; WTW = white-to-white; ECD = endothelial cell density.

**Table 2:** Endothelial cell density (ECD) loss in 3 time slots: early (preoperative vs. 6 months), late (6 months vs. last visit), and total (preoperative vs. last visit) (n = 176). The mean follow-up time was 40 ± 21 months (range: 12 to 92 months). Data are presented as mean, SD and 95% CI.

	Early	Late	Total ^1^	*p* ^2^
**ECD loss (cells/mm^2^)**	28 ± 121 (5, 51)	81 ± 163 (58, 104)	109 ± 169 (87, 132)	<0.014
**ECD loss (%)**	0.91 ± 4.28 (0.22, 1.59)	2.93 ± 5.92 (1.9, 3.97)	3.89 ± 6.11 (2.84, 4.93)	<0.01
**ECD loss rate per month (%)**	0.15 ± 0.71 (0.04–0.26)	0.06 ± 0.18 (0.03, 0.09)	0.10 ± 0.22 (0.07, 0.14)	<0.001
**ECD loss rate per year (%)**	1.81 ± 8.56 (0.43, 3.19)	0.68 ± 2.11 (0.34, 1.03)	1.21 ± 2.58 (0.83, 1.59)	

^1^ Note: Early and late ECD loss (%) are not directly additive; total ECD loss (%) was calculated independently from the underlying ECD measurements over the entire observation period. ^2^ LMM = linear mixed-effects models with Holm adjustment for multiple comparisons.

## Data Availability

The data presented in this study are available on request from M. Muzyka-Woźniak (mmw@spektrum.wroc.pl), as access is subject to legal considerations.

## Supplementary Materials

The following supporting information can be downloaded at: https://www.mdpi.com/article/10.3390/medsci14060656/s1, Supplementary Table S1. Preoperative biometric data for the subset of 176 eyes (95 patients) included in the analysis of ECD loss rates.

## Abbreviations

The following abbreviations are used in this manuscript:

ECD	Endothelial cell density
ICL	Implantable collamer lens
AL	Axial length
PD	Pupil diameter
ACD	Anterior chamber depth
AS-OCT	Anterior segment optical coherence tomography
WTW	White-to-white
CCT	Central corneal thickness
pIOLs	Phakic IOLs
FDA	Food and Drug Administration
LMM	Linear mixed-effects models
GEE	Generalized estimating equations
