# Supplementary material for: Short- and Long-Term Dynamics of Endothelial Cell Density and Vault Changes After Implantable Collamer Lens Implantation
Source: Med Sci (Basel). 2026 Oct 9;14(6):656. doi: 10.3390/medsci14060656 (PMC13706799; doi:10.3390/medsci14060656)
Supplement: Supplementary file 1 [file medsci-14-00656-s001.zip › medsci-4572223-supplementary.pdf]

Supplementary Table S1.

Preoperative biometric data for the subset of 176 eyes (95 patients) included in the analysis of ECD loss rates.

**ECD subset preoperative biometric data**				
**Parameter**	mean	SD	min	max
**AL (mm)**	26.55	1.28	24.19	30.42
**ACD (mm)**	3.27	0.23	2.78	3.90
**CCT (μm)**	537	38	450	653
**PD (mm)**	5.1	1.2	2.9	7.9
**WTW (mm)**	11.8	0.3	11.0	12.6
**Keratometric astigmatism (D)**	1.26	0.86	0.17	4.30
**ECD (cells/mm^2)**	2816	297	2015	3621

AL = axial length; ACD = anterior chamber depth; CCT = central corneal thickness; PD = pupil diameter; WTW = white-to-white; ECD = endothelial cell density.
